# Recurrence and Lethality in a Cohort of Minors With Suicidal Behaviour: 2010–2024

**DOI:** 10.62641/aep.v54i4.2219

**Published:** 2026-08-15

**Authors:** Marina Adrados-Pérez, Vicent Llorca-Bofí, Eugènia Nicolau-Subires, Laura Arenas-Pijoan, Teresa Jové-Miró, Andrea Jiménez-Mayoral, Diego de la Vega-Sánchez, María Mur-Laín, María Irigoyen-Otiñano

**Affiliations:** ^1^Psychiatry Service, Institut Pere Mata Hospital, 43206 Reus, Spain; ^2^Department of Psychiatry, University of Lérida, 25198 Lérida, Spain; ^3^Barcelona Clínic Schizophrenia Unit (BCSU), Neuroscience Institute, Hospital Clínic of Barcelona, August Pi i Sunyer Institute for Biomedical Research (IDIBAPS), 08036 Barcelona, Spain; ^4^Networked Biomedical Research Centre for Mental Health (CIBERSAM), Carlos III Health Institute,28029 Madrid, Spain; ^5^Department of Medicine, University of Barcelona, 08007 Barcelona, Spain; ^6^Psychiatry Service, Santa Maria University Hospital of Lérida, 25198 Lérida, Spain; ^7^Biomedical Research Institute of Lérida (IRB-Lérida), 25198 Lérida, Spain; ^8^Department of Psychiatry, University of Seville, 41004 Sevilla, Spain

**Keywords:** suicide attempts, lethality, recurrence, mortality, child and adolescent population

## Abstract

**Background:**

Suicidal behaviour in children and adolescents has increased significantly over the last decade, generating growing concern among healthcare professionals. However, clinical and evolutionary differences between minors and adults after a suicide attempt are not sufficiently defined. This study analyses the differential profile of minors treated for suicide attempts in a large provincial cohort to identify specific vulnerabilities and risk patterns.

**Methods:**

Retrospective cohort study (MCOSUL Cohort) that included all suicide attempts treated in provincial psychiatric emergency services between 2010 and 2024. Sociodemographic variables, psychiatric diagnoses, substance use, method and lethality of the index attempt, recurrence, and mortality were collected. High lethality was defined according to Beautrais criteria. χ^2^, Fisher, Student’s t, Kruskal–Wallis tests, and survival analyses for recurrence were applied.

**Results:**

Minors represented 8% of the cohort, with a female predominance (83%). They presented a higher prevalence of neurodevelopmental disorders and eating disorders (EDs), as well as a differential consumption pattern dominated by tetrahydrocannabinol (THC). They used more violent methods, especially cutting, without an increase in lethality. The Cox model shows that being a minor is significantly associated with earlier recurrence of suicidal behaviour (HR = 1.36), even after adjusting the analysis for sex, neurodevelopmental disorders, THC use, and the method employed. Adults showed higher lethality, mortality, and more frequent use of sedative medications as a suicide method. Recurrence of subsequent suicidal behaviour was similar but occurred earlier in minors.

**Conclusions:**

Adolescent suicidal behaviour appears to present a distinct clinical phenotype, characterized by a predominance of female adolescents, frequent neurodevelopmental vulnerabilities, THC use, low-lethality violent methods, and relatively rapid recurrence, highlighting the need for tailored prevention strategies.

## Introduction

Suicidal behaviour in children, adolescents, and young adults constitutes a highly relevant public health issue, with risk patterns that vary according to age, sex, psychiatric diagnosis, and substance use [1, 2, 3, 4]. In the past decade, suicide attempts and hospitalizations for suicidality in children and adolescents have significantly increased, reflecting an alarming trend in the mental health of the child and adolescent population [5]. It is estimated that around 18% of adolescents have seriously considered suicide, and approximately 6% have attempted it [6] (Suicide among minors has risen in recent decades, becoming one of the leading causes of death in this age group [7, 8], with suicide being the third leading cause of death among individuals aged 15 to 29 worldwide [8]. In recent decades, the lethality of suicide attempts in young people has shown a concerning increase: between 2% and 4% result in death [9, 10]). This rise is particularly notable in adolescents aged 15 to 19, with approximately a 30% increase in suicide rates over the last decades [11].

Recent meta-analytic evidence indicates that among individuals with mental disorders, suicide specifically contributes to a pooled of 8.31 (95% confidence interval (CI) 6.43–10.19) years of potential life lost (YPLL) [12]. In the United States, in 2018, an estimated 1,344,552 years of life were lost due to suicide, a figure comparable to the 1,591,487.5 years lost to COVID-19 in 2021 [13].

International evidence highlights that adolescents present an especially elevated risk of suicidal recurrence and of developing psychopathological and psychosocial complications following a first attempt [9, 14, 15, 16]. Among the most relevant risk factors are a family history of suicide, depression, anxiety, and substance abuse [17]. Additionally, a recent systematic review and meta-analysis showed that adolescents with suicidal ideation or previous attempts present a significantly higher risk of recurrence in early adulthood (odds ratio (OR) for attempt: 5.71; 95% CI: 2.40–13.61), as well as a greater likelihood of developing depressive and anxiety disorders [18]. Suicidal recurrence in adolescent and young populations has been widely documented [18, 19, 20]. A recent meta-analysis described that major repeaters (≥5 attempts across the lifespan) present a more severe clinical profile, with greater depressive severity, anxiety, substance abuse, aggressiveness, and hopelessness [21].

Longitudinal studies indicate that approximately one-quarter of adolescents who have attempted suicide repeat within the first year, and that the risk of recurrence increases with the number of prior attempts [22]. Specifically, a recent systematic review and meta-analysis focusing on adolescents and young adults (aged 10–24 years) included 4286 participants, identifying 1579 individuals with multiple attempts and 2707 with a single attempt; this results in a 37% prevalence of multiple attempters among those with at least one prior attempt [21].

It is worth noting that, according to final data published by the Instituto Nacional de Estadística (INE) [23] in December 2024, the suicide rate in Spain decreased in 2023 by 2.6% compared with 2022, with 4116 deaths by this cause. However, this reduction did not affect all age groups equally. In adolescents and young adults aged 15 to 29, there was a slight increase in cases (354, 13 more than in 2022). Therefore, although the overall suicide rate declined, suicides among adolescents did not decrease in 2023.

Thus, this study aims to analyse, over an extended observation period, the sociodemographic, clinical, and evolutionary variables of underage patients who attempted suicide and were treated in psychiatric emergency services between 2010 and 2024 in the only hospital providing urgent psychiatric care in the province, offering crucial epidemiological data to guide more specific prevention efforts.

## Materials and Methods

### Sample and Procedure

In this retrospective cohort study, participants come from the MCOSUL Cohort [24], which includes individuals who, after a suicide attempt, received treatment from the Liaison and Consultation Psychiatry team at the Arnau de Vilanova University Hospital and from the Psychiatric Emergency Service at the Santa María University Hospital, in Lérida, Spain, both sharing a single psychiatry department and serving an area with a population of 451,707 inhabitants [25].

The MCOSUL Cohort has an ambispective design, combining a retrospective review of clinical records with prospective follow-up. Retrospective data were obtained from medical records dating back to 2009, including sociodemographic and clinical information at the time of the index suicide attempt. Participants were subsequently followed up to record new suicide attempts or death until 2024.

### Variables

From the digital medical record, all patients treated for a suicide attempt—either in Psychiatric Emergency Services or in general emergency services—are collected. The following variables are recorded: sociodemographic (age, sex, occupation, academic level, household composition, marital status, etc.), clinical (diagnosis, prior follow-up, previous treatment, prior hospitalizations, personal or family history of suicidal behaviour, etc.), as well as variables related to the suicide attempt (method, lethality, and subsequent recurrence or death, indicating its date and cause). Psychiatric diagnoses were established by attending psychiatrists following the Diagnostic and Statistical Manual of Mental Disorders, Fourth Edition, Text Revision (DSM-IV-TR) criteria [26] as described in the cohort’s rationale paper [24].

Substance use was assessed through a two-fold methodology. First, recent consumption was objectively measured using toxicological urine analysis conducted at the time of the psychiatric emergency department visit. Second, substance use disorders (SUD) were diagnosed by attending psychiatrists according to DSM-IV-TR criteria [26], ensuring consistency with the overall diagnostic protocol of the cohort. This distinction is crucial, as a positive toxicology result (e.g., for tetrahydrocannabinol (THC)) indicates recent exposure but does not necessarily fulfill the diagnostic criteria for a chronic disorder [27].

### Statistical Analysis

Statistical analyses were conducted using IBM-SPSS v.23 (IBM Corp. IBM SPSS Statistics for Windows, Version 23.0. Armonk, NY, USA: IBM Corp; 2015). Continuous data were expressed as mean ± standard deviation, while categorical data were presented as percentages. Normal distribution was assessed using the Shapiro–Wilk test. For categorical variables, Chi-square test and Fisher’s exact test were used when appropriate. For continuous variables, Student’s t-test and the Kruskal–Wallis test were applied as parametric and non-parametric alternatives respectively. Comparisons of sociodemographic and clinical variables between minors and adults were performed. Survival analyses were conducted to assess time to recurrence of suicidal behaviour, using Kaplan-Meier curves. A Cox proportional hazards regression model was performed to identify variables independently associated with time to recurrence. The following covariates were included based on their clinical relevance and statistically significant differences between minors and adults in the univariate analyses: age group (minor vs. adult), sex, neurodevelopmental disorders, THC use, violent method, and high lethality. Type I error was set at the conventional value of 5% (alpha = 0.05) using a two-tailed approach.

To ensure specificity in the identification of the target event, suicide attempts (both index and follow-up) were defined according to Silverman [28]: “any self-inflicted, potentially harmful behaviour with a non-fatal outcome for which there is explicit or implicit evidence of intent to die”. This definition was used independently of ICD or DSM classifications for the behaviour itself. Following Beautrais’ criteria [29], suicide attempts requiring more than 24 hours of medical care in a general hospital were considered high lethality. Attempts requiring fewer than 24 hours were classified as low lethality. The Beautrais criteria were selected as an objective measure of medical lethality based on the duration of clinical care because they serve as an objective clinical measure that is widely used in retrospective studies of large cohorts to categorize the medical severity of attempts in a standardized manner [29]. Patients who reported 1 or 2 suicide attempts over the course of their lives (including the current one) were classified as “patients with no or low recurrence,” while those who reported more than two suicide attempts in total were classified as “patients with high recurrence”.

This classification was operationally defined to isolate “high-frequency repeaters” (those with ≥3 attempts) from occasional repeaters. The analytical purpose of this distinction is to identify the specific clinical and sociodemographic profile of patients with the highest density of suicidal events, who often require more intensive psychiatric monitoring.

Non-violent methods were defined as medication overdoses with sedative and/or non-sedative drugs. Violent methods included wrist-cutting, jumping from heights, hanging, and the use of weapons [30]. Age was grouped to facilitate interpretation: (1) young people under 24 years, (2) young adults aged 24–34, (3) middle-aged adults 35–65, and (4) older adults over 65 years. Finally, the sample was divided into four groups: low lethality and no recurrence (NONE), high lethality, high recurrence, and lethality plus recurrence (BOTH).

## Results

### Sociodemographic and Clinical Characteristics

Table 1 describes the sociodemographic characteristics of the study population. In the analysed sample, the child–adolescent population represented 8% of the total. When comparing gender distribution between groups, the proportion of females was significantly higher in the minor group (83%) than in the adult group (61%) (*p* < 0.001). In both populations, a female predominance was observed among those treated for suicide attempts. The mean age of underage patients treated was 15.30 years (SD 1.60), whereas in adults the mean age was 43.50 years (SD 15.10). The proportion of foreign-born individuals showed no significant differences (*p* = 0.182), although it was slightly higher in minors (20.20%) compared with adults (16%). Regarding diagnostic orientation after a suicide attempt, minors more frequently presented childhood-onset disorders (including Attention Deficit Hyperactivity Disorder (ADHD), autism spectrum disorders, and intellectual disabilities), as well as eating disorders (EDs). These differences were significant compared with the adult population. In contrast, the prevalence of anxiety–depressive disorders and personality disorders was comparable between groups. Substance-use analysis revealed that adults showed a higher prevalence of alcohol, cocaine, and polyconsumption, whereas in minors THC was the most frequently detected substance in urine tests.

**Table 1.** **Sample characteristics**.

Variables		Adults	Minors	*p*-value
		n = 1765 (92%)	n = 153 (8%)	
Female, n (%)		1077 (61)	127 (83)	<0.001***
Age, years (M±SD)		43.50 ± 15.10	15.30 ± 1.60	<0.001***
Foreign, n (%)		284 (16)	31 (20.20)	0.182
Psychiatric diagnosis, n(%)				
	Childhood-onset disorders	31 (1.70)	15 (9.70)	<0.001***
	Substance use disorder OH	48 (2.70)	0 (0)	0.018*
	Substance use disorder non-OH	202 (11.40)	2 (1.30)	<0.001***
	Psychosis	71 (4)	2 (1.30)	0.092
	Bipolar	20 (1.10)	0 (0)	0.186
	Unipolar	705 (39.90)	57 (37.20)	0.514
	Anxiety	113 (6.40)	13 (8.40)	0.316
	Adjustment	187 (10.50)	15 (9.80)	0.760
	ED	28 (1.50)	6 (3.90)	0.036*
	Impulse	15 (0.80)	4 (2.60)	0.059
	Personality	297 (16.80)	22 (14.30)	0.435
	Other	48 (2.60)	17 (11.10)	0.048*
Substance use, n (%)				
	Alcohol	679 (38.40)	28 (18.30)	<0.001***
	THC	167 (9.40)	30 (19.60)	<0.001***
	Cocaine	69 (3.90)	0 (0)	0.003**
	Heroin	6 (0.30)	0 (0)	0.607
	Polydrug	165 (9.30)	5 (3.20)	0.011*
Pathological gambling, n (%)		33 (1.80)	0 (0)	0.063

Note: OH, Alcohol; ED, eating disorder; THC, tetrahydrocannabinol. * Indicates statistical significance at a level of *p* < 0.05. ** Indicates statistical significance at a level of *p* < 0.01. *** Indicates statistical significance at a level of *p* < 0.001. Recurrence, lethality, and mortality.

### Recurrence, Lethality, and Mortality

Table 2 shows the lethality, recurrence, and mortality of patients who attempted suicide. Regarding recurrence during follow-up, both groups showed a similar pattern: for no recurrence, adults account for 32.2% and children for 34.6%; while for five or more additional attempts, adults account for 11% and children for 10.4%. No minor died by suicide or natural causes, whereas among adults, of the 106 individuals who died (6%), 38.6% died by suicide and 61.3% from other causes.

**Table 2.** **Lethality, recurrence, and mortality**.

Suicidal behavior		Adults	Minors	*p*-value
		n = 1765 (92%)	n = 153 (8%)	
Index episode lethal, n (%)		418 (23.60)	29 (18.90)	0.184
Total lethality, n (%)				
	1st lethal	187 (10.50)	18 (11.70)	0.653
	1st non-lethal & 2nd lethal	155 (8.70)	14 (9.10)	0.877
	Non-lethal & any lethal	161 (9.10)	6 (3.90)	0.014*
	Never lethal	1262 (71.50)	115 (75.10)	0.334
Previous recurrence, n (%)				
	None	839 (47.50)	98 (64)	<0.001***
	1 previous	497 (28.10)	39 (25.40)	0.480
	2–4 previous	345 (19.50)	15 (9.80)	<0.001**
	≥5 previous	84 (4.70)	1 (0.60)	0.006**
Recurrence during follow-up, n (%)				
	No recurrence	570 (32.20)	53 (34.60)	0.552
	1–2 recurrences	769 (43.50)	64 (41.80)	0.677
	≥3 recurrences	426 (24.10)	36 (23.50)	0.866
	≥5 recurrences	195 (11)	16 (10.40)	0.823
Deaths, n (%)		106 (6)	0 (0)	<0.001***
	Suicide	41 (38.60)	0 (0)	<0.001***
	Non-suicide	65 (61.30)	0 (0)	<0.001***

* Indicates statistical significance at a level of *p* < 0.05. ** Indicates statistical significance at a level of *p* < 0.01. *** Indicates statistical significance at a level of *p* < 0.001.

Table 3 shows the characteristics of patients who presented high recurrence (≥3 suicide attempts) after the index suicide attempt. Marked differences were observed by age. The percentage of females was significantly higher among minors, reaching 97.2%, whereas in adults females represented only 30.9%. The proportion of foreign-born patients was similar between groups, with 16.6% in minors and 10.6% in adults. In the high-recurrence subgroup (≥3 attempts), minors showed a significantly higher prevalence of adjustment disorder (13.8% vs. 3.1%, *p* = 0.009) and THC use (30.5% vs. 10.6%, *p* = 0.002) compared to adults. In contrast, the diagnosis of non-alcoholic substance use disorder was significantly less frequent in minors (2.8%) than in adults (15.5%) (*p* = 0.021). Regarding substance use, THC was associated with recurrence among minors, whereas in adults the associated factors were alcohol use and polyconsumption. No significant differences were observed regarding gambling disorder. Total lethality during the observation period showed that recurrent minors tended to engage in subsequent attempts of greater severity.

**Table 3.** **Significance test of stepwise regression model**.

Variables		Adults	Minors	*p*-value
		n = 426 (24.10%)	n = 36 (23.50%)	
Female, n (%)		294 (69)	35 (97.20)	<0.001***
Foreign, n (%)		45 (10.60)	6 (16.60)	0.194
Psychiatric diagnosis, n (%)				
	Childhood-onset disorders	9 (2.10)	3 (8.30)	0.059
	Substance use disorder OH	17 (4.00)	0 (0)	0.245
	Substance use disorder non-OH	66 (15.50)	1 (2.80)	0.021*
	Psychosis	19 (4.50)	0 (0)	0.207
	Bipolar	8 (1.90)	0 (0)	0.407
	Unipolar	209 (49.10)	19 (52.70)	0.668
	Anxiety	25 (5.90)	1 (2.70)	0.710
	Adjustment	13 (3.10)	5 (13.80)	0.009**
	ED	10 (2.30)	2 (5.50)	0.238
	Impulse	3 (0.70)	1 (2.70)	0.278
	Personality	45 (10.60)	3 (8.30)	0.472
	Other	2 (0.40)	1 (2.80)	0.057
Substance use, n (%)				
	Alcohol	185 (43.50)	9 (25)	0.031*
	THC	45 (10.60)	11 (30.50)	0.002**
	Cocaine	24 (5.60)	0 (0)	0.135
	Polydrug	46 (10.80)	0 (0)	0.020*
Pathological gambling, n (%)		11 (2.60)	0 (0)	0.406
Index episode lethal, n (%)		93 (21.80)	4 (11.10)	0.091
Total lethality, n (%)				
	1st lethal	14 (3.30)	0 (0)	0.269
	1st non-lethal & 2nd lethal	21 (4.90)	6 (16.60)	0.004**
	Non-lethal & any lethal	124 (29.10)	6 (16.60)	0.111
	Never lethal	267 (62.70)	24 (66.60)	0.634

Note: This table presents the sociodemographic and clinical characteristics of patients who made three or more suicide attempts during follow-up. This subgroup includes patients with five or more recurrences (adults: n = 195; minors: n = 16), as these are nested categories. Percentages refer to the total sample of adults (n = 1765) and minors (n = 153) respectively. OH, Alcohol; ED, eating disorder; THC, tetrahydrocannabinol. * Indicates statistical significance at a level of *p* < 0.05. ** Indicates statistical significance at a level of *p* < 0.01. *** Indicates statistical significance at a level of *p* < 0.001.

**Fig. 1.**
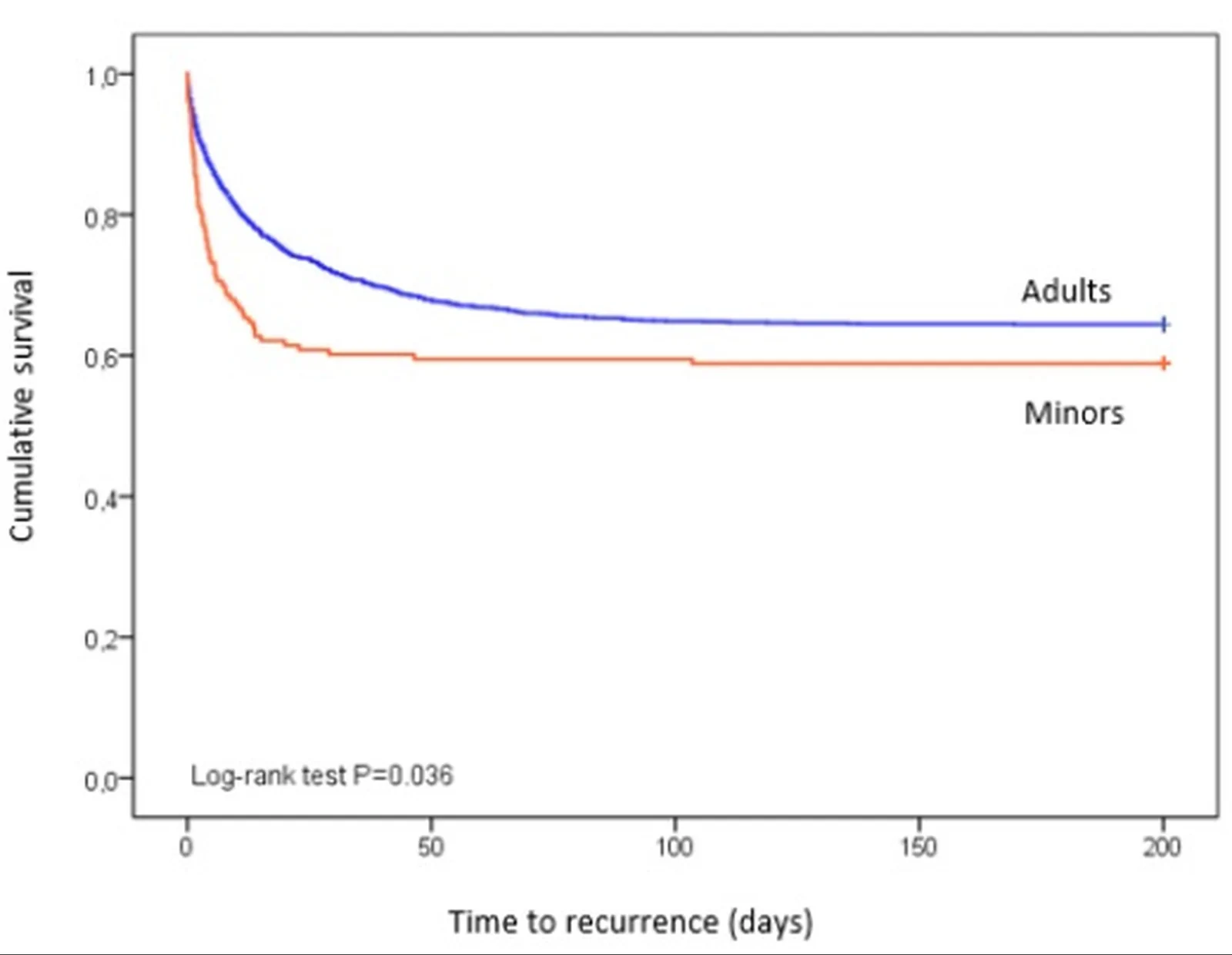
**Kaplan–Meier survival analysis for time from the index episode to recurrence comparing adults and minors**.

Fig. 1 presents the survival analysis from the index suicide attempt to the next suicidal event. It shows that minors take less time to reattempt suicidal behaviour compared with adults. In the Cox proportional hazards model adjusted for sex, neurodevelopmental disorders, THC use, violent method, and high lethality, being a minor was associated with earlier recurrence of suicidal behaviour (hazard ratio (HR) = 1.36, 95% CI: 1.01–1.85, *p* = 0.036).

### Suicide Methods

Table 4 shows the suicide methods used in the attempts. Analysis of the method used in the index attempt revealed that adults more frequently used sedative medications, while no significant differences were found in the use of non-sedative medications or drugs between groups. Firearm use was recorded in 10 adults (0.6%) and 4 minors (2.6%). Minors also showed a greater tendency to use cutting as a suicide method, while the prevalence of other violent methods—including defenestration, drowning, self-immolation, and hanging—was comparable between groups. Despite minors using significantly more violent methods, no differences were found in medical lethality or clinical severity (as measured by hospital stay). It is crucial to note that low medical lethality does not necessarily equate to low psychological severity or intent. The prevalence of visits for suicidal ideation was similar in both groups, at approximately 25%.

**Table 4.** **Suicide method in index attempt**.

Suicide method, n (%)	Adults	Minors	*p*-value
	n = 1765 (92%)	n = 153 (8%)	
Sedative drugs/substances	771 (43.70)	51 (33.30)	0.013*
Non-sedative drugs/substances	318 (18)	31 (20.30)	0.276
Firearms	10 (0.60)	4 (2.60)	0.021*
Self-immolation	1 (0.10)	0 (0)	0.920
Drowning	6 (0.30)	0 (0)	0.607
Cutting	94 (5.30)	23 (15)	<0.001***
Defenestration / Jumping	67 (3.80)	4 (2.60)	0.318
Hanging	31 (1.80)	1 (0.70)	0.261
Suicidal ideation	447 (25.30)	38 (24.80)	0.491
Other (ingestion, objects, accidents)	18 (1)	1 (0.70)	0.545
Unknown	2 (0.10)	0 (0)	0.847

* Indicates statistical significance at a level of *p* < 0.05. ** Indicates statistical significance at a level of *p* < 0.01. *** Indicates statistical significance at a level of *p* < 0.001.

## Discussion

Suicidal behaviour in the child and adolescent population presents specific clinical and psychosocial characteristics that clearly differentiate it from adults. In our sample, minors attended after a suicide attempt represented 8% of the total, with a female predominance (83% vs. 61% in adults) and a mean age of 15.3 years vs. 43.5 years. They showed a higher prevalence of childhood-onset disorders and EDs, while depression, anxiety, and personality disorders were similar in both groups. Substance use clearly differentiated the groups: alcohol, cocaine, and polysubstance use in adults, THC in minors. Adolescents used more cutting and firearms, although with lower lethality, whereas adults used sedative drugs, with higher lethality.

Recurrence was faster in minors, although the overall percentage of repeat attempters was similar between adults and minors. High-recurrence profiles showed clear differences: minors were characterized by a predominance of females, THC consumption, and adjustment disorders, whereas in adults, alcohol use, polyconsumption, and depressive or personality diagnoses were more prominent. Suicidal ideation was similar (∼25%) and no minor died, compared with 106 deaths in adults, 38.6% by suicide. However, the absence of observed deaths in the minor group should be interpreted with caution due to the sample size (n = 153), as it does not imply an absence of mortality risk in this population. These findings reflect clear differences by age, sex, diagnosis, and consumption, highlighting adolescence as a period of critical vulnerability.

In our sample, we found among minors attended after a suicide attempt a clearly higher female prevalence in the adolescent population (83% vs. 61%), which is consistent with the international literature, which highlights the predisposition of adolescent girls to ideation and non-lethal attempts, while lethality tends to predominate in adult men [31, 32].

Numerous international studies show that suicide risk increases progressively throughout adolescence and reaches its maximum in young adults, with lower rates in preadolescent children [1, 2, 33]. This finding places adolescents at the peak of vulnerability for suicide attempts and coincides with international data showing an increase in the incidence of attempts between ages 10 and 17 [3, 31, 34].

In our sample, the proportion of foreign patients was slightly higher in minors (20.2%) than in adults (16%), but this difference did not reach statistical significance. This suggests that, in our context, immigration is not a relevant differential predictor of suicidal behaviour, in line with observation from countries with similar demographic patterns [35]. It is worth considering the demographic characteristics of the province of Lérida, where according to data from the Statistical Institute of Catalonia [25], in January 2023 there were 88,356 people of foreign nationality, representing approximately 19.75% of the total.

According to the literature, the profile of the minor patient with a suicide attempt is characterized by a female predominance, adolescence, the presence of depressive disorders, ADHD, and emotional difficulties inherent to development, as well as a significant influence of family and school factors [1, 36]. An emerging consumption of THC, the use of low-lethality methods, and early recurrence are also described in the literature [37, 38]. Our results coincide with this pattern: female predominance, greater presence of childhood-onset disorders and EDs, THC use, low-lethality methods, and rapid recurrence.

In our sample, minors showed a higher prevalence of childhood-onset disorders and EDs while the prevalence of unipolar depression, anxiety, and personality disorders was similar in both groups. This reflects that the psychiatric factors associated with ideation and suicidal behaviour are shared in both populations, but disorders such as ADHD, autism spectrum disorders, and EDs constitute more relevant factors in minors, in agreement with international findings [3, 4, 32].

Previous studies show that substance use increases the risk of suicide and recurrence [39, 40]. The literature shows that in adults, alcohol, cocaine, and polysubstance use predominate [41], whereas in adolescents, THC is more strongly associated with ideation and suicide attempts [42, 43]. Our data reflect this as well, showing that in minors with high recurrence. Our results specifically demonstrate that THC use was particularly relevant, while in adults alcohol, cocaine, and polysubstance use predominated.

International studies describe a consistent pattern: adolescents use more violent but less lethal methods, whereas adults, especially men, use more lethal methods such as firearms [34, 44, 45]. A striking finding in our cohort was a “lethality paradox” among minors: despite using more violent methods, their attempts showed low medical severity. Cutting was the most representative example (15% vs. 5.3%), a method frequently linked to emotional dysregulation and impulsivity rather than high suicidal intent. This overlap with non-suicidal self-injury underscores the importance of careful clinical assessment in adolescents. Notably, firearm use was more prevalent among minors than adults (2.6% vs. 0.6%), a concerning finding given its high lethality potential that reinforces the relevance of means restriction strategies in this age group [46, 47]. In contrast, adults more frequently used sedative drugs (43.7% vs. 33.3%). This pattern is consistent with the literature, which suggests that methodological violence in adolescents does not necessarily translate into mortality [1, 44, 45]. However, this should be considered a hypothetical explanation or possible mechanism rather than a definitive conclusion, as our data do not include direct measures such as injury location, weapon type, or failed attempts. It may be attributed to possible mechanisms such as impulsivity, low lethal intent, or limited planning. In adults, high-lethality methods were associated with higher mortality, consistent with established definitions integrating method potential and objective circumstances of the attempt [48].

The literature shows differences by gender and age in patterns of recurrence and lethality in suicide attempts, with higher frequency of attempts and recurrence in young adults, and increased lethality from age 45 onward [49]. In minors, the history of previous attempts is lower, and recurrence occurs more rapidly if they do not receive specialized intervention [1, 2, 33]. In our cohort, minors had fewer antecedents but recurred in a shorter time. While our model adjusts for several clinical phenotypes, the accelerated recurrence in minors may be deeply influenced by unmeasured psychosocial factors, such as family instability or school-related stress, which were not available in our database.

The finding of accelerated recurrence in minors has direct clinical implications for post-emergency management. Standard follow-up periods may be insufficient for this vulnerable group [50]. We propose the implementation of “rapid response” protocols, ensuring a clinical contact (either in-person or via tele-psychiatry) within 48–72 hours after the emergency department visit. This early intervention should prioritize three core pillars: first, the review and reinforcement of safety plans; second, the active restriction of access to lethal means [46], which is particularly critical given the high prevalence of violent methods like cutting and firearms observed in our underage sample; and third, the early detection of relapse symptoms [37]. A well-established model for this approach is the Catalonia Suicide Risk Code (CRS) [51, 52]. According to Pérez *et al*. [53] the CRS immediate follow-up actions include a face-to-face appointment within 10 days for adults and 72 hours for minors, as well as a telephone contact at 30 days of enrolment. While this model has proven effective in our region, its applicability to other healthcare systems would require careful adaptation to local resources and clinical workflows.

Among patients with high recurrence (≥3 attempts) after the index attempt (24.1% adults, 23.5% minors), minors were mostly female, with predominance of THC use and adjustment disorders, while adults showed predominance of alcohol, polysubstance use, and major depression, according to findings by other researchers [43, 54, 55]. We found that overall lethality was higher in adults, although some subsequent attempts in recurrent minors reached greater severity. These findings underscore consistent differences by gender, age, and type of diagnosis in the recurrence and lethality of suicide attempts [3, 31, 45]. Due to the sample imbalance, these results should be replicated in larger, more balanced multicenter studies to confirm the stability of the age-related hazard.

In summary, these findings suggest an association between underage status and a pattern of rapid recurrence; however, this should be interpreted as a regional clinical phenotype rather than a stable independent effect. Unmeasured psychosocial factors, such as family instability or school-related stress, likely contribute to this pattern, and the observed sample imbalance (reflecting the real-world epidemiology of our health region where minors represent a smaller proportion of psychiatric emergencies) may also influence the results. Therefore, further studies in larger and more diverse cultural and healthcare contexts are needed to assess the generalizability and stability of these patterns.

### Limitations and Strengths

This study presents several limitations that must be carefully considered. First, the retrospective, single-center design and the inclusion of only emergency department cases introduce selection bias; this may overestimate clinical severity while underestimating out-of-hospital suicide mortality and milder cases. Second, the study could not control for unmeasured psychosocial confounders, such as socioeconomic status, family functioning, or specific post-emergency interventions, and lacked standardized psychometric scales to assess subjective suicidal intent.

Third, the use of Beautrais criteria and our specific recurrence threshold (≥3 attempts) may reflect hospital resource utilization patterns rather than intrinsic biological severity or standard epidemiological definitions. Finally, the small sample size and imbalance of the minor subgroup (8%) limits statistical power for rare events and may affect the stability of the multivariable Cox regression estimates. Consequently, these findings should be interpreted as a regional clinical phenotype with limited generalizability to different healthcare systems.

Despite these constraints, the study’s strengths include a robust 14-year observation period, a large overall sample size, and a standardized characterization of clinical and evolutionary variables, providing rare longitudinal data on suicidal behavior in the Spanish youth population.

## Conclusions

Suicidal behaviour in the underage population presents a characteristic profile that differs from that of adults. In our setting, this profile is characterized by a predominance of females, a higher prevalence of psychiatric diagnoses with childhood onset, and clinical features such as EDs and THC use. Suicide attempts in minors tend to involve more violent methods, although with lower clinical lethality compared to adults, and are associated with a shorter time to recurrence after the first episode.

## Availability of Data and Materials

The analytical data used in this study are available from the corresponding author upon reasonable request.
